# Removal of Micrometer Size Morphological Defects and Enhancement of Ultraviolet Emission by Thermal Treatment of Ga-Doped ZnO Nanostructures

**DOI:** 10.1371/journal.pone.0086418

**Published:** 2014-01-28

**Authors:** Umair Manzoor, Do K. Kim, Mohammad Islam, Arshad S. Bhatti

**Affiliations:** 1 Alamoudi Water Chair, King Saud University, Riyadh. Kingdom of Saudi Arabia; 2 Center for Micro and Nano Devices, Department of Physics, COMSATS Institute of Information Technology, Islamabad. Pakistan; 3 Department of Materials Science and Engineering, Korea Advanced Institute of Science and Technology (KAIST), Daejeon. Republic of Korea; 4 College of Engineering, King Saud University, Riyadh, Saudi Arabia; King Abdullah University of Science and Technology, Saudi Arabia

## Abstract

Mixed morphologies of Ga-doped Zinc Oxide (ZnO) nanostructures are synthesized by vapor transport method. Systematic scanning electron microscope (SEM) studies of different morphologies, after periodic heat treatments, gives direct evidence of sublimation. SEM micrographs give direct evidence that morphological defects of nanostructures can be removed by annealing. Ultra Violet (UV) and visible emission depends strongly on the annealing temperatures and luminescent efficiency of UV emission is enhanced significantly with each subsequent heat treatment. X-Ray diffraction (XRD) results suggest that crystal quality improved by annealing and phase separation may occur at high temperatures.

## Introduction

The study of different doped and undoped ZnO nanostructures are of great interest in the scientific community owing to their excellent optical, electrical, gas sensing and piezoelectric properties [1]–[4]. Doped ZnO is also one of the most explored materials for practical applications i.e. sensors, transparent conductor oxide (TCO), and photocatalysis etc [5], [6]. Some of these applications require high electrical conductivity, which may be achieved by replacing Zn^2+^ ions with higher valence ions. These ions act as efficient shallow donors and In^3+^, Al^3+^ and Ga3+ ions have shown better properties than others [7], [8]. In gallium-doped zinc oxide (Ga-doped ZnO), Ga3+ is expected to cause a small lattice distortion (similar radii sizes of Zn and Ga) and at the same time is an efficient shallow donor in ZnO. However, Ga doping may produce ZnGa2O4 phase when the doping exceed a certain limit. Solubility limits are reported to be close to 3 at% of Ga in ZnO [9], [10]. Literature is limited and a comprehensive study is required on the effect of annealing on Ga-doped ZnO nanostructures. This will be important for high temperature applications i.e. gas sensors in which usual operating temperatures are more than 400°C [11].

In this report, we demonstrate, for the first time that micrometer size defects in Ga-doped ZnO nanostructures can be removed by carefully tuning the annealing conditions. Phase separation also occurs at 900°C. Direct evidence of sublimation, at temperatures much lower than the synthesis temperatures, is also provided and possible reasons for the improvement in optical and structural properties are discussed.

## Experimental Procedure

Ga-doped ZnO nanostructures were synthesized by vapor transport method. Equal amounts (by weight) of ZnO powder (99.0%, HAYASHI PURE CHEMICALS INDUSTRIES, Osaka, Japan) and carbon black were mixed for 4 hours in a ball mill. 0.15 g Ga_2_O_3_ was added in 0.6 g of the mixture, mixed using mortar and pasture (source mixture) and loaded into an alumina boat. A Silicon (Si) substrate was placed on top of alumina boat. The boat was then placed at the center of the tube furnace. Ga-doped ZnO nanostructures were synthesized at 950°C with 15 minutes holding time. Ar was used as carrier gas and flow rate of Ar and O_2_ was 150 and 4 sccm respectively. Morphology was characterized by using scanning electron microscopy (XL30 PHILIPS Netherlands) fitted with Energy Dispersive Spectroscopy (EDX) for elemental analysis. Phase analysis of the deposited nanostructures was done by using x-ray diffraction (XRD RIGAKU Tokyo, Japan). Room temperature photoluminescence of the nanostructures was measured using Xenon lamp with the excitation wavelength of 325 nm.

Post-synthesis heat treatment was done by first transferring nanostructures on a Si substrate coated with thin layer of SiO_2_. Small amount of ethanol was dropped on the nanostructures and dried in air to ensure better dispersion and sticking of nanostructures to the substrate. Figure S1 is the low magnification SEM image of the substrate. The figure clearly suggests that nanostructures are dispersed on the substrate. Post-synthesis heat treatment of these nanostructures was done by heating the same substrate subsequently at 600°C, 700°C, 800°C and 900°C for 1 hour in O_2_ (99.999% pure, flow rate = 25 sccm). SEM, XRD and photoluminance (PL) were measured after every heat treatment.

## Results and Discussion

**Figure 1:**
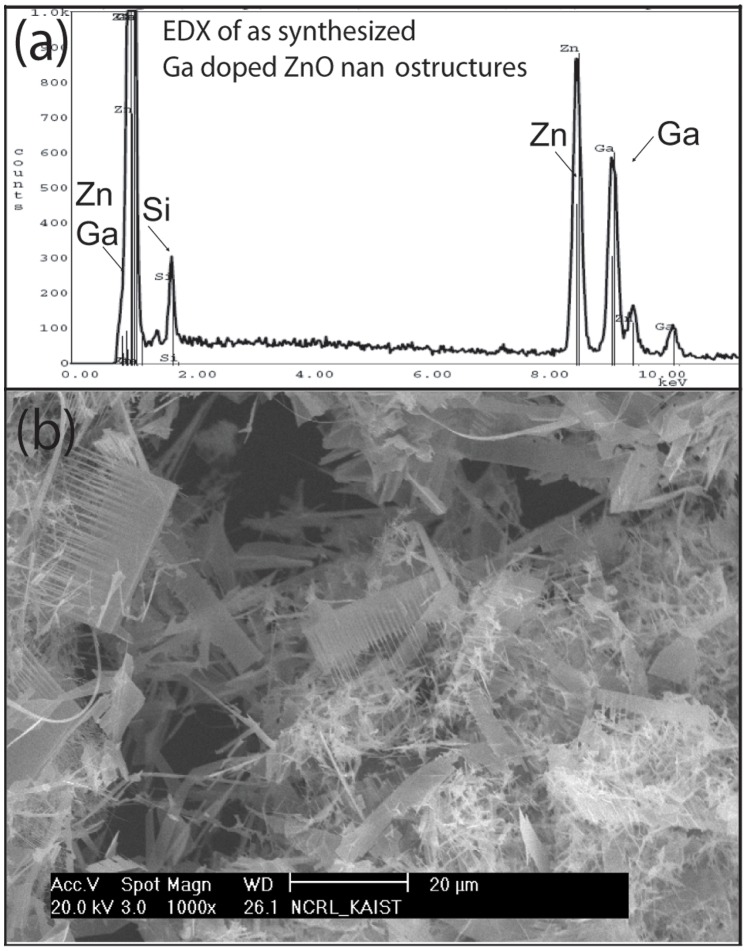
EDX spectra of Ga-doped ZnO nanostructures (a) clearly suggest presence of significant amount of Ga. Si peak may come from the substrate. (b) Low magnification SEM image clearly suggested presence of mixed morphologies. Nanocombs, Nanobelts and nanowires can be clearly seen on the substrate.

Small part of the as-deposited powder was transferred to a substrate and SEM, XRD and PL properties were investigated after each subsequent heat treatment at 600°C, 700°C, 800°C and 900°C for 1 hour. Figure 1(a) is the typical EDX area scan of Ga-doped ZnO nanostructures. The results clearly shows primary and secondary peaks of Zn and Ga, suggesting that significant amount of Ga is present. A distinct Si peak is also present which comes from Si substrate. Figure 1(b) is the low magnification image of Ga-doped ZnO nanostructures after transferring on other substrate. The results clearly show different morphologies i.e. comb-shape nanostructures, Nanobelts, nanosheets and nanowires etc. These structures are unique in the sense that there are very few reports on mixed morphologies of Ga-doped ZnO nanostructures [12].

**Figure 2:**
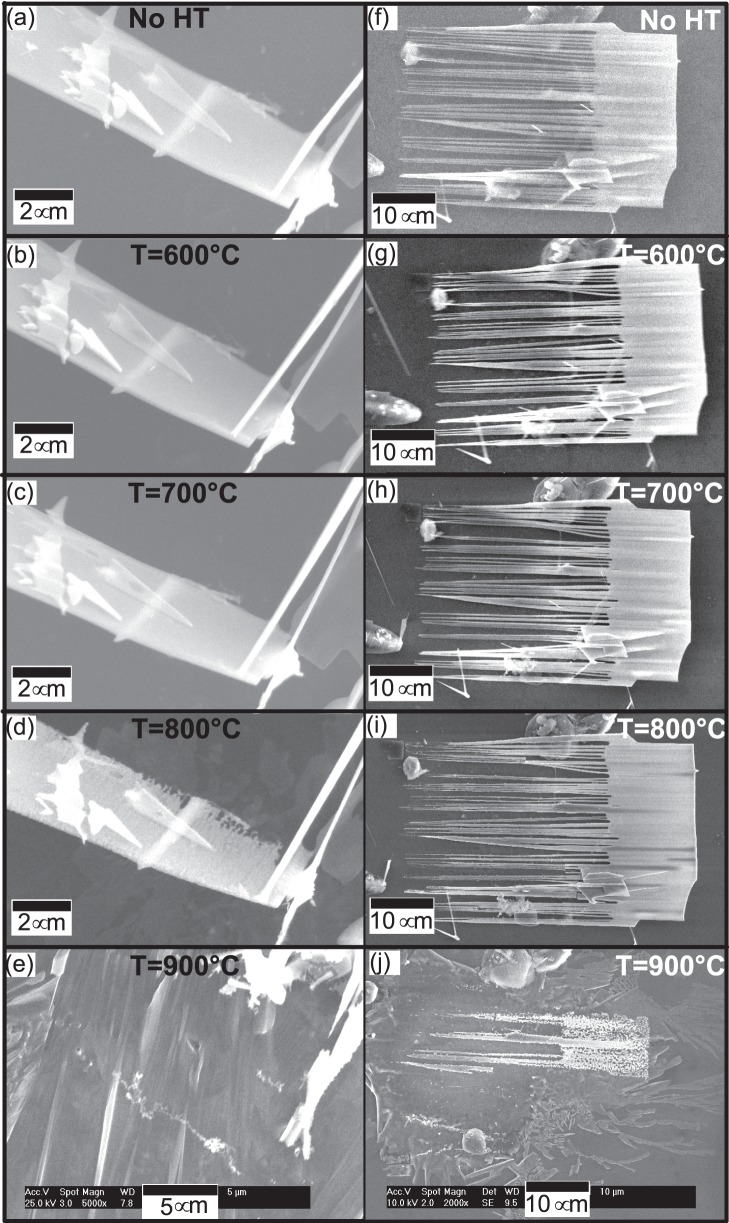
SEM images of (a∼e) nanosheet (f∼j) nanocomb, after subsequent heat treatments at 600°C, 700°C, 800°C and 900°C. The results clearly suggest that these nanostructures are thermally stable till 700°C. There is a significant degradation after 800°C and nanostructures completely disappeared after heat treatment at 900°C.

Figure 2(a∼f) shows SEM micrographs of ZnO sheet shape structure without any heat treatment and after thermal annealing at 600°C, 700°C, 800°C and 900°C respectively. There is no significant difference in morphology after heating at 600°C and 700°C. SEM micrographs clearly suggest a systematic degradation with temperature after annealing at 800°C and 900°C. Morphological changes are visible and the sheet-shape structure is converted into one sided saw-shape structure at 800°C. The other side only has a rough surface suggesting uneven thermal etching behavior on both sides. There can be many possible explanations for this and O_2_ flow, uneven thickness and crystal structure can play a significant role. After heating the same substrate at 900°C, actual structure disappeared and some new structures (may be due to heterogeneous nucleation on the existing ZnO rods on the upper left corner) were developed.

Figure 2 (f∼j) shows SEM micrographs of ZnO comb-shape structure without any heat treatment and after thermal annealing at 600, 700, 800 and 900°C respectively. SEM micrographs clearly suggest a systematic degradation with temperature, showing no significant difference after heating at 600°C and 700°C. Morphological degradation of the nanometer sized secondary arms begins at 800°C and the free ends of the secondary arms are etched out. At this stage, the thick primary sheet-shape structure has no significant difference. However, the general comb-shape structure still persists after annealing at 800°C. When the same sample was heated to 900°C, the actual comb-shape structure disappeared and only an inconsistent thick film was visible in the micrograph.

Zinc oxide sublimes congruently by decomposition to the gaseous elements according to the following reaction
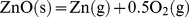
(1)


In our case, as annealing temperature is raised, sublimation rate increases. During high temperature (>700°C) annealing, the surface morphology of bulk ZnO is affected by the evaporation of lattice constituents and surface becomes rough due to the continuous evaporation [13], [14]. As the temperature further increases (900°C), both ZnO nanostructures are completely etched out and only nanoparticles are left. Previous reports suggested that prominent sublimation in bulk ZnO occurs at 1100°C [15]. It is interesting to note that sublimation in nanostructures occurred at 800°C which is much lower than the synthesis temperature (950°C). Figure 2 gives direct evidence that ZnO nanostructures sublimes at much lower temperatures than the synthesis temperature. It may be because of the nanosize of these structures.

**Figure 3:**
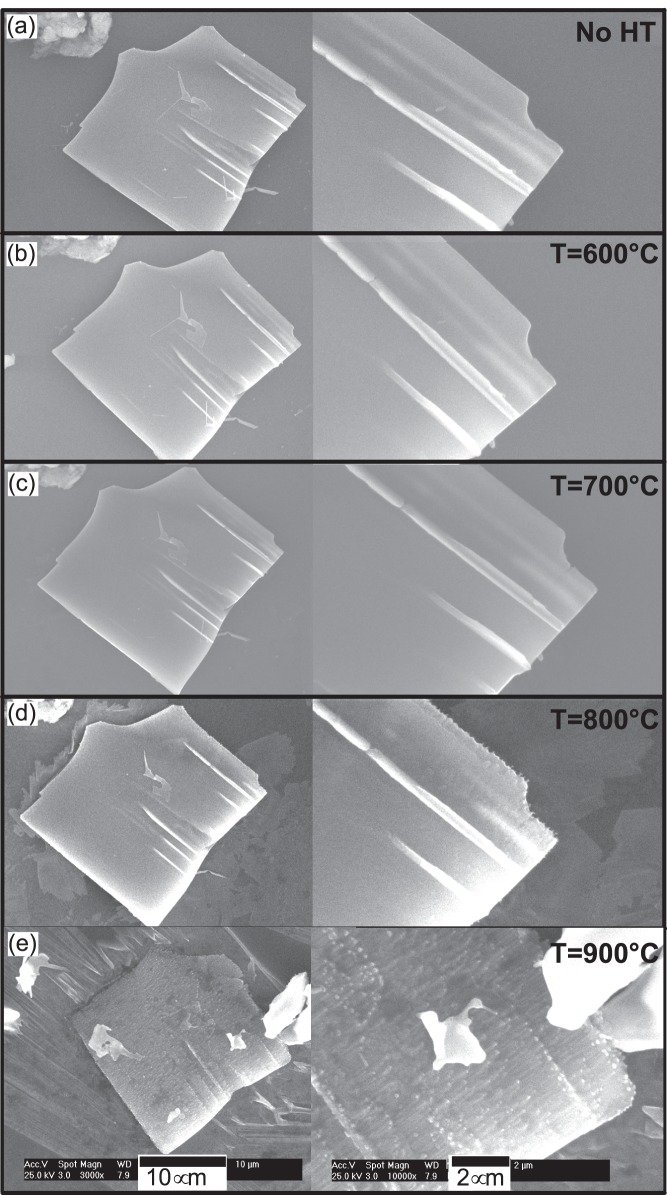
SEM micrograph of a sheet-shape structure after subsequent heat treatments at 600°C, 700°C, 800°C and 900°C. Slight degradation starts at 800°C and surface became rough. After heating the sheet-shape structure at 900°C, the morphological defects (mentioned as 1, 2, 3 & 4) are filled. This is a unique result and the change is attributed to the condensation of Zn species due to supersaturation.

Figure 3 is the series of SEM images of another sheet-shape structure on the same substrate with 2 different magnifications. This structure does not have regular edges and have morphological defects. The results without any heat treatment and after thermal annealing at 600, 700 and 800°C are very similar to Figure 2, i.e. there are no morphological changes after annealing at 600°C and 700°C and surface became rough (thermal etching/sublimation) at 800°C.

Very interesting and different results are observed after annealing at 900°C (Figure 3-e). The morphology changes are visible in the SEM micrograph at 4 different points. At points 1 and 2, sharp edges are etched out and became smooth. At points 3 there was a morphological defect which was completely etched out. At point 4 there is a near “L” shape morphological effect approximately 2 µm in length. There is no change in the overall morphology after subsequent annealing at 600, 700 and 800°C. After heating the same structure at 900°C the “L” shape defect is completely filled to an extent of perfection. SEM micrographs of all nanostructures with focus on the discussed defects (high magnification images) are shown in Supplementary Figure S2.

### Growth Mechanism

The overall growth for this type of defect removal can be divided into 4 steps: (i) Source species generation, (ii) Transport of source species to the high energy surface i.e. defect site (iii) Impingement of material on the surface i.e. condensation and (iv) Incorporation of material into the nanostructure i.e. diffusion. Thermodynamically, necessary-but not necessarily sufficient-conditions are degree of supersaturation and the chemical potential of different species of Zn and O. Also, it was well-known that different planes of ZnO nanostructures have different energies. The polarity and high energy of ZnO (0 0 01) surface can be the key enabling factor to determine the nanostructures grown [16].

Wurtzite ZnO is a polar crystal, having a hexagonal unit cell with nonpolar faces capped by polar (0001) and (0001) basal planes [17]. Polar faces with surface dipoles are thermodynamically less stable than nonpolar faces, often undergoing rearrangement to minimize their surface energy [18], [19]. It is established and calculations of the partial pressures and degree of saturation of Zn vapor over condensed phase Zn show that while the partial pressure of Zn vapor is relatively large, the vapor is under-saturated and so will not condense on the surface unless energetically suitable accommodation sites exist for nucleation to take place. Therefore, the self-catalyzed process (at high energy defect sites) is likely the nucleation mechanism for the filling of nanostructure defects. Once the nanostructure has nucleated and started to grow, Zn vapor atoms will readily condense at the ZnO crystallite, and react with O_2_ to form ZnO. Over growth, in this situation is possible for very high super-saturated conditions. However, over growth is not visible in our case.

**Figure 4:**
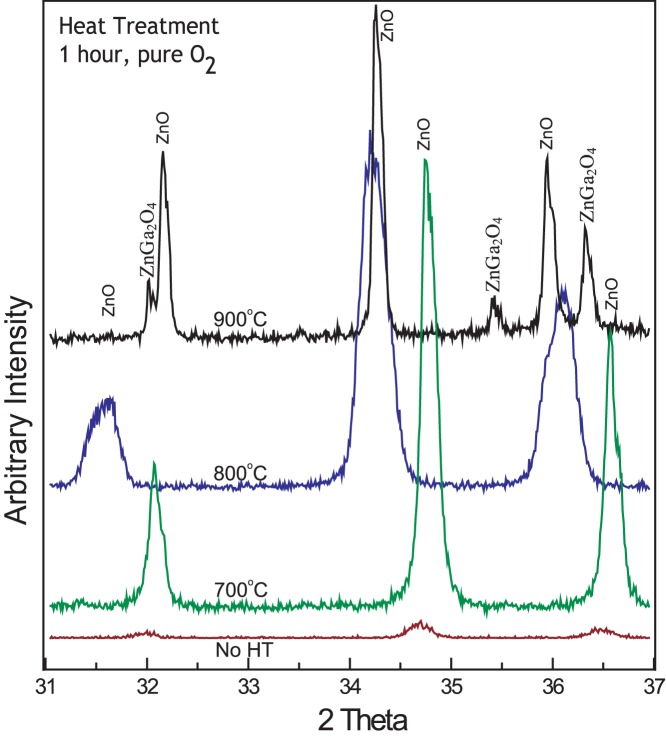
Comparison of XRD results of Ga-doped ZnO nanostructures without any heat treatment and after annealing at 700°C, 800°C and 900°C. XRD spectra are direct observation of narrower FWHM after annealing, suggesting better crystalinity. New peaks appear after annealing at 900°C, suggesting that a new phase is formed.

The evolution of XRD results with different annealing temperatures is shown in Fig. 4. XRD results clearly suggest change in peak positions and full width at half-maximum (FWHM) after subsequent heat treatment. XRD results of as synthesized Ga-doped ZnO nanostructures showed very low intensity peaks, suggesting low crystalinity and high defect density. When the same substrate was heated at 700°C there is a peak shift and narrower FWHM. Also, only ZnO peaks are present, and no peaks of other phases appear on the graph. The peak shift and narrow FWHM may be due to high annealing temperatures which help to enhance the mobility of atoms, subsequently resulting in reduced defect concentration and improve crystal quality [16]. Doping and defect concentration are related and the amount of defects/doping influences the “*d*” spacing of crystal. This effect can be clearly seen in the intensity and shifts in the XRD peaks. In Figure 4 peaks shift is random i.e. after annealing at 700°C the shift is towards higher angle but after annealing at 800°C the shift is towards lower angle. A reasonable explanation is that this drastic change in “*d*” spacing can be because of different types of defects and defect concentrations after annealing at high temperatures.

Similar results were obtained when the same substrate was heated at 800°C, except that FWHM is now broader. Very interesting and different results are observed after annealing at 900°C. FWHM of ZnO peaks again become narrower and new peaks of ZnGa_2_O_4_ are clearly visible in the graph. The angular peak position of bulk crystalline ZnO with (101) orientation is 2θ = 36.3° which is comparable to JCPDS card # 65–3411.

The changes in XRD patterns with subsequent heat treatments reveal some interesting findings. As synthesized sample initially has low crystalinity. This may be due to Ga doping and/or other crystal defects. It is established that deep-level defects in ZnO can be recovered by annealing the material at high temperatures [20] and this fact is reflected in the XRD pattern of sample annealed at 700°C. Point defects and other defects are mobile at these temperatures. After annealing the same sample at 800°C, the high amount of Ga in the lattice may starts dissolution which may be the prominent mechanism at this stage, resulting in distortion of crystal, decrease in XRD intensity and broadening of FWHM. At 900°C, thermal energy was enough for rejection of excess Ga and phase separation occurs. It is well-known that ZnGa_2_O_4_ is formed when ZnO and Ga are mixed together in appropriate conditions [21]. This is reflected in the XRD patterns and peaks of ZnGa_2_O_4_ are clearly visible in the XRD patterns. In short, XRD patterns suggests that defect density decreases in the initial annealing phase (till 700°C), then again start increasing (800°C) and further heat treatment (900°C) results in phase separation.

**Figure 5:**
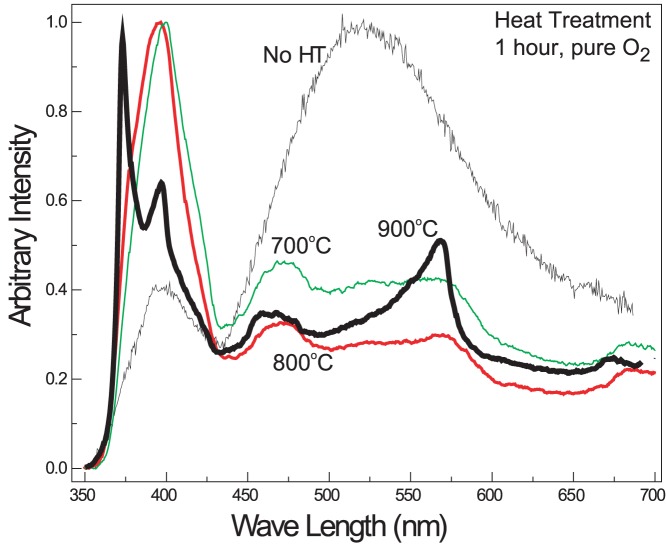
Room temperature PL of nanostructures was measured using Xe lamp (excited wavelength: 325 nm). PL spectra are a clear indication of increase in UV intensity with respect to green intensity after heat treatment at 700°C. Also, the broad DLE peak is replaced by 2 smaller peaks. This is an indication that the defects type and density is changed after annealing. After annealing at 900°C, NBE peak also splits into 2 peaks at 380 nm and 395 nm which may be the result of formation of ZnGa_2_O_4_ phase.

PL spectrum of the substrate was measured at room-temperature using Xe lamp (325 nm) as excitation source. PL peaks of as-synthesized Ga-doped ZnO. nanostructures (Figure 5, No HT) mainly consists of a weak UV emission and a strong green emission. The UV emission, located at 400 nm, is the exciton recombination related to near-band edge emission (NBE) of ZnO and the deep-level emission (DLE) at 525 nm usually results from the radiative recombination of a photogenerated hole with an electron occupying the oxygen vacancy and other defects [22]. Defects are often electrically active and introduce levels in the band gap of the semiconductor, which involve transitions between different charge states of the same defect [23]. Optical properties of ZnO can be tuned by annealing the samples in different environments [24]. We annealed our sample under O_2_ atmosphere at 600, 700, 800 and 900°C in pure oxygen environment. The results suggest that UV intensity (NBE peak) significantly increases after annealing at 700°C. DLE peak clearly split into two peaks at 475 nm and 575 nm. These two peaks are clear indication that defect density and types and drastically changed after annealing at 700°C and only specific type of defects are present. Information about atomic diffusion or migration of point defects in ZnO is currently limited. Activation energies of zinc self-diffusion, in pure ZnO were reported to be in a range from 1.9 to 3.3 eV, while activation energies for oxygen self-diffusion were reported to span a much wider range, from 1.5 to 7.5 eV [25]. Doping also effect these values and interpreting these results or using them in a predictive manner is not straightforward. After annealing the same sample at 800°C, there is a blue shift of NBE peak and peak intensities of DLE peaks decreases. Point defects i.e. oxygen vacancy, oxygen interstitial, zinc vacancy, and impurities are considered to be possible origins for these bands [26]. It is well known that DLE related defects cannot be completely removed by annealing and, on the contrary, the annealing conditions actually favor their formation. Point defects at compound semiconductor surfaces are, for entropy reasons, thermodynamically stable at high temperatures [27]. Therefore it is difficult to remove completely the point defects only by thermal treatment in Ga-doped ZnO nanostructures and a minor peak may always present in the PL data.

When the same sample was annealed at 900°C, there is again a drastic change in the PL spectra (as in XRD, Figure 4). NBE peak splits into 2 very distinct peaks at 380 nm and 395 nm. DLE peak didn’t show significant shift but their relative intensity increased to significant levels. These phenomena of NBE peak splitting can be explained by comparing XRD results in which peaks of other phase are clearly visible. It is expected that ZnGa_2_O_4_ is precipitated out as separate phase. The two separate peaks in the UV range can be from the 2 distinct phases appearing after annealing at 900°C.

XRD results and PL data are in perfect agreement with each other. The increase in UV and XRD intensities suggests decrease in the crystal defects and improves quality of ZnO. XRD peak shift also indicates an improvement in the overall crystal structure. Hence it can be suggested that high annealing temperatures (upto 800°C) provide enough energy to enhance mobility and diffusion/rejection that could decrease the defects and improve overall crystal quality [28]. However, when Ga-doped ZnO nanostructures were annealed at 900°C phase separation occurs and new peaks appear in XRD and PL data.

Previous researchers have suggested that defects may degrade the performance of optical devices fabricated from III-V semiconductors [29]. Ko et al find a correlation between UV intensity and threading dislocations present in ZnO epilayer, suggesting that UV intensity increases with decrease in threading dislocation concentration [16]. Two different groups in independent studies concluded that after annealing the ZnO films, UV peak increases significantly, indicating that quality of ZnO films were improved through annealing [26]. However, according to our knowledge, this is the first report which suggests that removal/patch-up of micrometer size morphological defects is possible along with the enhancement of ultraviolet emission and better crystalinity (based on XRD results) by thermal treatment of Ga-doped ZnO nanostructures.

## Conclusion

Different morphologies of Ga-doped ZnO nanostructures were synthesized by vapor transport method. Elemental analysis confirmed the presence of Ga while XRD results only showed ZnO peaks for as synthesized nanostructures. SEM micrographs suggested a systematic morphological degradation of different nanostructures after annealing at 800°C and 900°C. However, some of the micrometer size morphological defects/irregularities were filled after high temperature annealing. According to our understanding, this is the first ever scientific proof (SEM micrographs) that high energy morphological defects/irregularities of nanostructures can be removed by post-synthesis annealing. XRD and PL results supported each other, indicating that crystal quality gradually improves with subsequent heat treatment, which may be attributed to the higher atomic mobility, resulting in reduced defect concentrations.

## Supporting Information

Figure S1 **Low magnification SEM image of Ga-doped ZnO nanostructures after transferring on Si substrate coated with a thin layer of SiO_2_.**
(TIF)

Figure S2 **Series of SEM micrographs of different morphologies of Ga-doped ZnO nanostructures after subsequent heat treatments at 600°C, 700°C, 800°C and 900°C for 1 hour in O_2_ (99.999% pure, flow rate = 25 sccm).** SEM images same nanostructures (different magnifications) are also part of the paper as Figure 2 and Figure 3.
(TIF)

## References

[1] Law M, Goldberger J, Yang P (2004) Semiconductor Nanowires and nanotubes. Annu Rev Mater Res 34: 83–122.

[2] Chen Z, Wu N, Shan Z, Zhao M, Li S, et al (2005) Effect of N2 flow rate on morphology and structure of ZnO nanocrystals synthesized via vapor deposition. Scripta Mater 52: 63–67.

[3] Kolmakov A, Moskovits M (2004) Chemical sensing and catalysis by one-dimensional metal-oxide nanostructures. Annu Rev Mater Res 34: 151–180.

[4] Simon H, Krekeler T, Schaan G, Mader W (2012) Metal-Seeded Growth Mechanism of ZnO Nanowires. Cryst Growth Des 13: 572–580.

[5] Cheong KY, Muti N, Ramanan SR (2002) Electrical and optical studies of ZnO:Ga thin films fabricated via the sol-gel technique. Thin Solid Films 410: 142–146.

[6] Amin M, Manzoor U, Islam M, Bhatti A, Shah N (2012) Synthesis of ZnO Nanostructures for Low Temperature CO and UV Sensing. Sensors 12: 13842–13851. 23202024 10.3390/s121013842 PMC3545595

[7] Assuncao V, Fortunato E, Marques A, Goncalves A, Ferreira I, et al (2003) New challenges on gallium-doped zinc oxide films prepared by r.f. magnetron sputtering. Thin Solid Films 442: 102–106.

[8] Fortunato E, Goncalves A, Assuncao V, Marques A, Aguas H, et al (2003) Growth of ZnO:Ga thin films at room temperature on polymeric substrates: thickness dependence. Thin Solid Films 442: 121–126.

[9] Roberts N, Wang R-P, Sleight AW, Warren WWJ (1998) Al and Ga impurity nuclear magnetic resonance in ZnO:Al and ZnO:Ga. Phys Rev B: Condens Matter 57: 5734–5741.

[10] Wang R, Sleight AW, Cleary D (1996) High Conductivity in Gallium-Doped Zinc Oxide Powders. Chem Mater 8: 433–439.

[11] Gu F, Fen Wang S, Feng Song C, Meng KL, Xin Qi Y, et al (2003) Synthesis and luminescence properties of SnO2 nanoparticles. Chem Phys Lett 372: 451–454.

[12] Oh SJ, Jung MN, Ha SY, Choi SG, Kim JJ, et al (2008) Microstructure evolution of highly Ga-doped ZnO nanocrystals. Physica E 41: 31–35.

[13] Kohl D, Henzler M, Heiland G (1974) Low temperature sublimation processes from clean cleaved polar surfaces of zinc oxide crystals during first heating. Surf Sci 41: 403–411.

[14] Grunze M, Hirschwald W, Hofmann D (1981) Zinc oxide: Surface structure, stability, and mechanisms of surface reactions. J Cryst Growth 52, Part 1: 241–249.

[15] Iwanaga H, Yoshiie T, Yamaguchi T, Shibata N (1979) Crystal growth and sublimation in II-VI compounds along their polar axis. J Cryst Growth 47: 703–711.

[16] Manzoor U, Kim DK (2006) Synthesis and enhancement of ultraviolet emission by post-thermal treatment of unique zinc oxide comb-shaped dendritic nanostructures. Scripta Mater 54: 807–811.

[17] Fang ZB, Yan ZJ, Tan YS, Liu XQ, Wang YY (2005) Influence of post-annealing treatment on the structure properties of ZnO films. Appl Surf Sci 241: 303–308.

[18] Peterson RB, Fields CL, Gregg BA (2004) Epitaxial Chemical Deposition of ZnO Nanocolumns from NaOH Solutions. Langmuir 20: 5114–5118. 15984276 10.1021/la049683c

[19] Greene LE, Yuhas BD, Law M, Zitoun D, Yang P (2006) Solution-Grown Zinc Oxide Nanowires. Inorg Chem 45: 7535–7543. 16961338 10.1021/ic0601900

[20] Mtangi W, Auret FD, Diale M, Meyer WE, Chawanda A, et al (2012) Effects of high temperature annealing on single crystal ZnO and ZnO devices. J Appl Phys 111: 084503–084503–084506.

[21] De Souza Goncalves A, Antonio Marques de Lima S, Rosaly Davolos M, Gutierrez Antonio S, de Oliveira Paiva-Santos C (2006) The effects of ZnGa2O4 formation on structural and optical properties of ZnO:Ga powders. J Solid State Chem 179: 1330–1334.

[22] Vanheusden K, Warren WL, Seager CH, Tallant DR, Voigt JA, et al (1996) Mechanisms behind green photoluminescence in ZnO phosphor powders. J Appl Phys 79: 7983–7990.

[23] Lannoo M. In: Bourgoin, J (1983) Point defects in semiconductors. Springer Berlin, New York. Vol 2, Chapter 3.

[24] Lim J, Shin K, Kim HW, Lee C (2004) Effect of annealing on the photoluminescence characteristics of ZnO thin films grown on the sapphire substrate by atomic layer epitaxy. Mater Sci Eng B 107: 301–304.

[25] Janotti A, Van de Walle CG (2007) Native point defects in ZnO. Phys Rev B: Condens Matter. 76: 165202.

[26] Zhang Y, Du G, Yang X, Zhao B, Ma Y, et al (2004) Effect of annealing on ZnO thin films grown on (001) silicon substrate by low-pressure metalorganic chemical vapour deposition. Semicond Sci Technol 19: 755–758.

[27] Gopel W (1979) Initial steps of interface formation: Surface states and thermodynamics. J Vac Sci Technol 16: 1229–1235.

[28] Lin BX, Fu ZX, Jia YB (2001) Green Luminescent Center in Undoped Zinc Oxide Films Deposited on Silicon Substrates Appl Phys lett. 2001, 79, 943–45.

[29] Ko H-J, Yao T, Chen Y, Hong S-K (2002) Investigation of ZnO epilayers grown under various Zn/O ratios by plasma-assisted molecular-beam epitaxy. J Appl Phys 92: 4354–4360.

