# Supplementary figures and images for: Removal of Micrometer Size Morphological Defects and Enhancement of Ultraviolet Emission by Thermal Treatment of Ga-Doped ZnO Nanostructures
Source: PLoS One. 2014 Jan 28;9(1):e86418. doi: 10.1371/journal.pone.0086418 (PMC3904914; doi:10.1371/journal.pone.0086418)

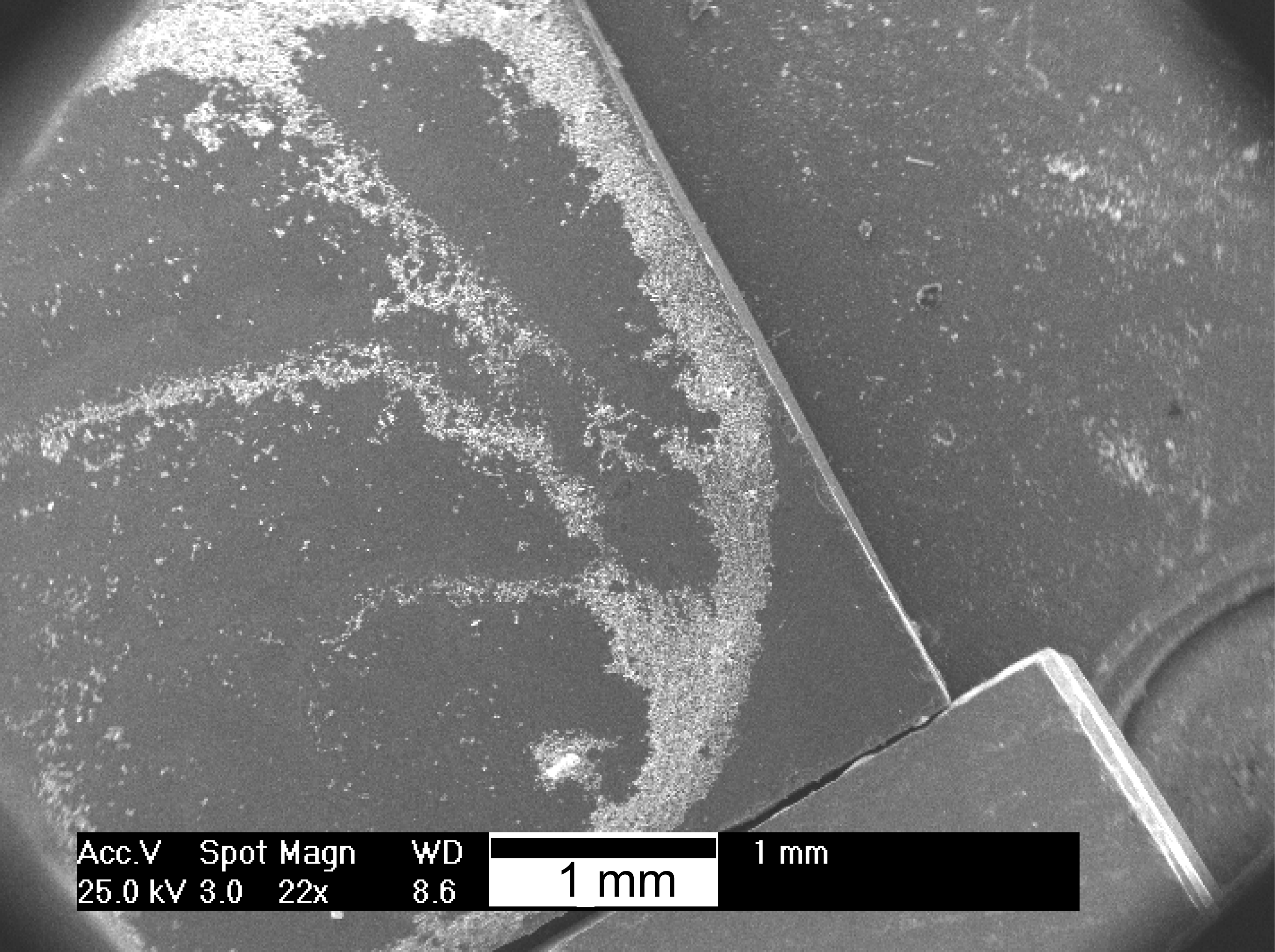

Supplement: Figure S1 — Low magnification SEM image of Ga-doped ZnO nanostructures after transferring on Si substrate coated with a thin layer of SiO2. (TIF) [file pone.0086418.s001.tif]

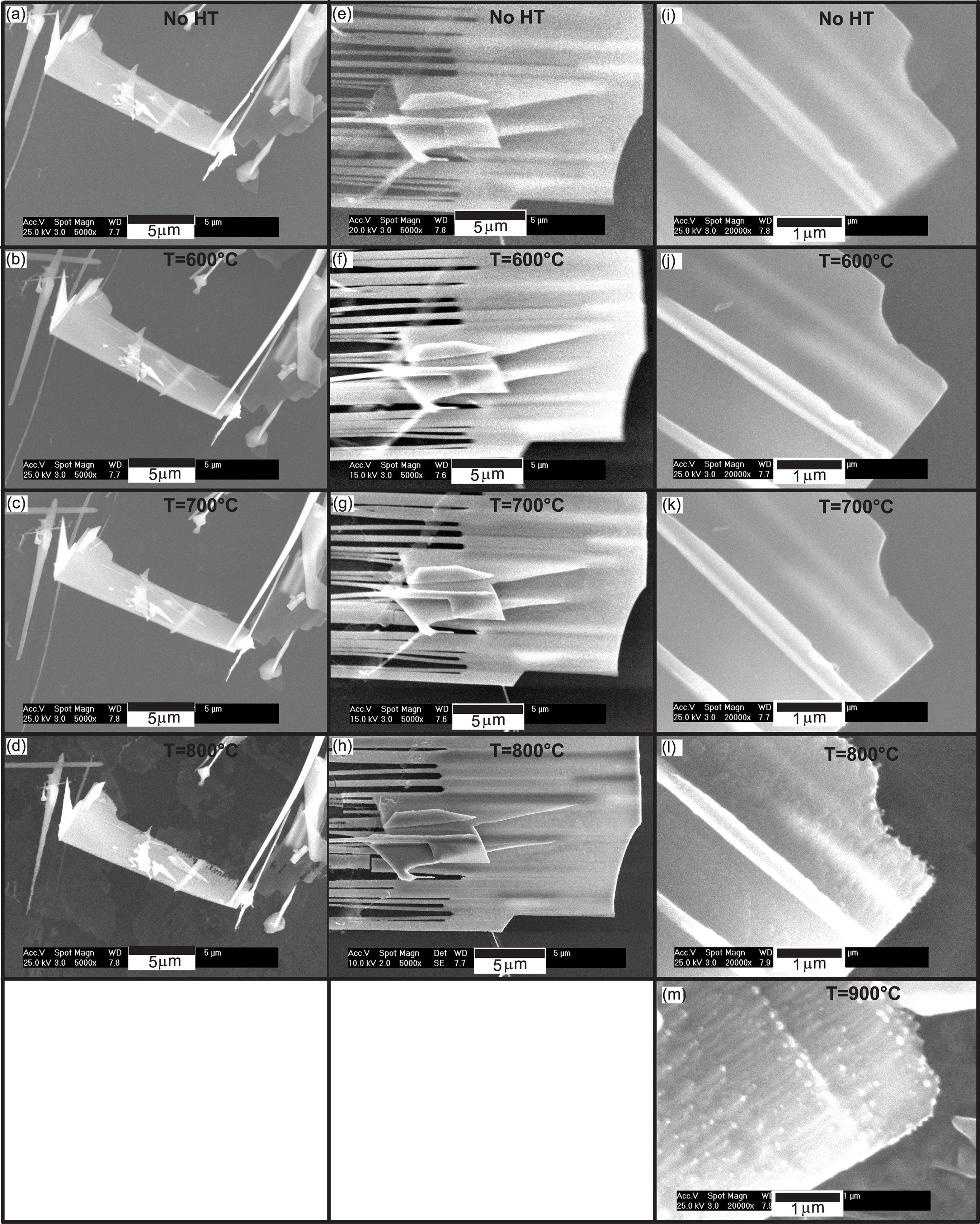

Supplement: Figure S2 — Series of SEM micrographs of different morphologies of Ga-doped ZnO nanostructures after subsequent heat treatments at 600°C, 700°C, 800°C and 900°C for 1 hour in O2 (99.999% pure, flow rate = 25 sccm). SEM images same nanostructures (different magnifications) are also part of the paper as Figure 2 and Figure 3. (TIF) [file pone.0086418.s002.tif]
